# Infant Cognitive Development and Stimulating Parenting Practices in Rural China

**DOI:** 10.3390/ijerph18105277

**Published:** 2021-05-15

**Authors:** Hannah Johnstone, Yi Yang, Hao Xue, Scott Rozelle

**Affiliations:** Stanford Center on China’s Economy and Institutions, Stanford University, 616 Jane Stanford Way, Stanford, CA 94305, USA; hannahfj@stanford.edu (H.J.); atticay@stanford.edu (Y.Y.); rozelle@stanford.edu (S.R.)

**Keywords:** infant and toddler cognitive development, infant and toddler stimulation, stimulative parenting, rural China

## Abstract

This study examines the prevalence of cognitive delay among infants and toddlers in rural China and its relationship with one of the potential sources of the observed delay: low levels of stimulating parenting practices (SPPs). Data were compiled from five distinct studies, resulting in a pooled sample of 4436 caregivers of 6–29-month-old infants. The sampling sites span five provinces in rural China. According to the data, on average, rates of delay are high—51 percent. The low rates of SPPs among our sample demonstrate that this may be one source of the high prevalence of delays. The results of the multivariate regression analysis reveal that reading books and singing songs are each significantly associated with an increase in infant cognitive score by 1.62 points (*p* = 0.003) and 2.00 points (*p* < 0.001), respectively. Telling stories to infants, however, is not significantly associated with infant cognitive scores. Our findings indicate that caregivers with different characteristics engage in various levels of stimulating practices and have infants with different rates of delay. Specifically, infants of better-educated mothers who have greater household assets are in families in which the caregivers provide more SPPs and have infants who score higher on the study’s cognitive abilities scales.

## 1. Introduction

Early childhood development research in developed countries has increasingly focused on measuring the relationship between stimulating parenting practices (SPPs) and childhood development, with many studies finding positive associations between SPPs and the development of the basic cognitive functions of their children [1]. The majority of this research has been conducted in subpopulations in developed countries, using samples from relatively affluent and healthy populations (with relatively low percentages, or normal shares, of populations with cognitive delays). These studies have shown that physical and mental stimulation are crucial to the development and growth of cognitive, language, social-emotional, and motor skills in children and infants [2,3,4].

Less research on this topic has been done in developing nations. Worldwide, it is estimated that nearly 40% of children and infants are not reaching their potential for development [5], the majority of who are located in low-and-middle-income countries (LMICs). Although less research has been conducted in LMICs, the available literature similarly shows that quality parent-child engagement in the form of physical and verbal interactions has positive and significant associations with executive functions and performance intelligence in infants [6,7]. Moreover, childhood developmental outcomes have been shown to predict higher academic performance, future earnings for individuals, and greater national economic growth of populations [8].

While the developmental studies referred to in the previous paragraph show that research on early childhood development has been conducted in LMICs, relatively few studies that examine the nature of development outcomes of infants have been conducted in rural China, despite the fact that a substantial percentage of China’s population lives in rural provinces. It is estimated that approximately 70% of all infants in China are born and reside in rural provinces or live in migrant communities in the suburbs of China’s cities [9].

Recent literature measuring infant cognitive delay in rural China has found that rates of cognitive delay during infancy are high and parental engagement in stimulating parenting practices is low [10,11]. These individual studies, however, were only conducted in relatively isolated communities (geographically) and are limited in sample size. Therefore, based on any one of the papers, it is difficult to draw general conclusions about infant development in rural China or the relationship between infant cognitive development and stimulating parenting practices.

There also has been scarce research that seeks to analyze the characteristics of the families and caregivers who are (and are not) engaging in SPPs either internationally or in rural China. In the international literature, a small set of studies focuses on identifying family characteristics associated with low levels of SPPs. One study determined that there was a significant positive relationship between caregiver educational attainment and SPPs [12]. Ghosh et al. (2015) identified a positive relationship between socioeconomic status (SES) and SPPs [13]. Obradovic et al. (2016) found that when the primary caregiver was the child’s mother (rather than the child’s grandmother or other caregiver types), the intensity of SPPs rose [7]. To our knowledge, however, no study in rural China has identified and analyzed the factors associated with poor SPPs as one of its main focal points.

The overall goal of our paper is to measure the prevalence of development delays in rural China and then to identify the factors and outcomes associated with SPPs across multiple provinces in rural China. To achieve this goal, our study has four objectives. First, we determine the prevalence of cognitive delay in our sample areas. Next, we document the prevalence of SPPs (drawing on questions derived from the Family Care Index) across rural China. Third, we investigate the associations between different SPPs and cognitive development within our samples. Finally, we measure the relationship between family characteristics (characteristics of caregivers, their households, and infants) and the frequency and type of SPP.

## 2. Data and Methods

### 2.1. Pooled Dataset

In this section, we describe the characteristics of the studies that are pooled and used in this study, as well as give a brief description of each individual study. Next, we provide an overview of the sample selection processes, followed by a description of the data collection and survey instruments that were utilized. Finally, we present the statistical analysis used in this paper as well as explain the models that were implemented to examine the data.

The data used in this study were aggregated from five studies across China and will be referred to as the pooled dataset. Every study included in the pooled dataset used the same measures to assess cognitive outcomes, using the Bayley-III scale (with the exception of study 1, which used the Bayley-I MDI scale) and SPPs (reading books, singing songs, and telling stories). Taken together, the pooled dataset included observations on caregiver-infant/toddler dyads from five provinces, 34 counties, and 4436 individuals. A summary of the five studies with eight study sites and corresponding publications is presented in Appendix A Table A1.

### 2.2. Individual Datasets

Study 1 was carried out in Shaanxi, and data collection was conducted during a four-week period in 2014. Household surveys were administered to the caregivers of 525 randomly selected infants and toddlers. The data collection effort covered 131 villages across four nationally designated poverty counties in southern Shaanxi Province. In 2015, research teams carried out study 2 (Yunnan and Hebei), for which surveys were administered to 440 caregiver-infant/toddler dyads who were living in 43 villages across two designated poverty counties (one in each of the study provinces). The second Shaanxi study (study 3) was conducted in 2016. Data collection teams administered surveys to 1832 randomly selected households in 100 villages across 22 nationally designated poverty counties. Study 4 was carried out in 2017, for which surveys were conducted in three provinces, Beijing, Henan, and Shaanxi. Although a share of the families who were surveyed was chosen from subpopulations that were living in urban migrant communities, all of the respondents were rural residents (had rural hukou). In total, 625 caregiver-infant/toddler dyads were selected across the three provinces. The final dataset (study 5) was collected in Yunnan in 2019. Research teams administered surveys to 1014 randomly selected caregiver-infant/toddler dyads across 224 villages in two townships located in a nationally designated poverty county in Yunnan.

### 2.3. Sample Selection

Studies 1, 2, 3, and 5 used nearly identical four-step sampling protocols to select sample households for the individual studies. First, the research teams selected nationally designated poverty counties. Second, the research teams gathered a list of all townships, excluding those that housed the county seat (as those tend to be more urbanized) and those without villages with a population of 800 or more. Next, the sample villages were randomly selected. Finally, the local bureau of health provided lists of households with infants and toddlers in each of the sample villages in the desired age range. From those lists, the teams selected all infants and toddlers within the village.

The sample selection for study 4 varied somewhat from the other studies, although the targeted infants and toddlers were the same (that is, they were from families with rural hukou). In counties of Henan province, sample towns and villages were randomly chosen from a comprehensive list of all towns and villages. Next, the researchers randomly chose households with infants and toddlers aged 6 to 30 months. To obtain a sampling from Beijing and Shaanxi, the research team visited urban government offices (the metropolitan Family Planning offices) and obtained lists of neighborhoods that were known to house migrant communities with rural hukou. From this list of neighborhoods, the researchers randomly selected migrant households with infants aged 6 to 30 months.

While it is true that the sample migrant households from study 4 were living in migrant communities (and not in rural villages), all sample families in this study had rural hukou, i.e., rural residency status [14]. Although relatively little is known about ECD in rural migrant communities, studies have found that migrant children have similar scores on cognitive scales when they are school-aged [15,16]. Given these two sets of findings, we believe that the migrant sample of infants is suitable for our purposes of examining rates of cognitive delay.

### 2.4. Data Collection and Survey Instruments

Although the data are aggregated from multiple surveys, all five surveys used similar data collection strategies. In each survey, questionnaires were distributed by local undergraduate and graduate students who were recruited from academic departments by the research teams relevant to the content of the survey. All students underwent comprehensive, multi-day training for each survey.

The first survey block collected information on the cognitive development of sampled infants, using the Bayley Scales of Infant and Toddler Development, Third Edition (BSID-III). Enumerators attended a week-long training course on how to administer the BSID-III, which included a two and a half day experiential learning program in the field. Accompanied by their primary caregiver, infants completed a standardized series of developmental tasks (which included items such as attention to familiar and unfamiliar objects, looking for a fallen object, and pretend play) to assess their performance as related to information processing, counting, and number skills [17]. The test also considered the infant’s gestational and chronological age. This test is listed by the American Psychiatric Association as a standard way to diagnose certain developmental disorders and has been translated and adapted to the Chinese context. The BDSI-III scale used in China has been tested for inter-item consistency, test-retest reliability, and inter-rater reliability, and it has been shown to be reliable and valid [17].

The BSID-III data allowed the research teams to measure the rates of cognitive delay in the samples. Cognitive delay was defined according to documented distributions of BSID scores in reference populations. In a healthy population, the mean cognitive score is 105 with a standard deviation (SD) of 9.6 [18]. Mild delay is defined as a score between one and two SDs below the mean. An infant is considered to have mild cognitive delay if the infant scores between 85.8 and 95.4 (inclusive). A moderate or severe delay is defined as a score that is more than two SDs below the mean. Infants with scores below 85.8 are considered moderately or severely delayed. In this study, cognitive delay (in general) is defined as a score below 95.4 [18].

In the second part of the survey, enumerators collected additional survey data from the sample households. For this part of the survey, enumerators underwent a three-day training course on how to administer household surveys, after which all enumerators participated in a two day pilot program in non-sampled villages to practice survey presentation. During the survey, enumerators presented each household with a standardized survey questionnaire and read aloud each question as it was written. For example, to assess a household’s SES, enumerators asked families whether they possessed each of the following ten items: a flush toilet, running water, car, motorcycle, refrigerator, water boiler, laundry machine, computer, internet, and air conditioning. These questions were adapted from the Family Care Indicators (FCI) survey. The FCI has been shown to have adequate test-retest reliability. Previous studies have shown significant relationships between FCI and cognitive development in LMICs [19]. This indicator has been translated and adapted for use in the context of rural China [20,21]. Subsets of FCI measures are commonly used in studies associated with child development [22,23,24,25,26].

The last survey block collected demographic and socioeconomic information from all sample households. This included each infant’s gender and age and whether the infant was born prematurely. The data collected also contained socioeconomic information, including whether the primary caregiver was the mother, mother’s educational attainment, mother’s age, household SES, and whether the household received a rural subsistence allowance. In this study, household SES was determined using an asset-based measure. Enumerators asked families whether they possessed each of the following ten items: a flush toilet, running water, car, motorcycle, refrigerator, water boiler, laundry machine, computer, internet, and air conditioning. For each item, a dummy variable was generated to indicate an absence or possession of each of the listed items, where 0 means absence and 1 means possession. SES was an interval measure obtained by adding the values of each of those 10 measures and running a principal component analysis to generate a standardized composite scale from −0.53 to 2.53, with an SD of 1 as a proxy for family asset index (the higher the number, the higher the SES).

### 2.5. Statistical Analysis

Our statistical analysis is comprised of three parts. First, we run descriptive statistics of our samples and break them down by province to show the prevalence of cognitive scores, cognitive delays, and SPPs across rural communities. To examine the prevalence of cognitive delay and SPPs, we first report descriptive statistics from the pooled dataset as a whole. Following, we report the prevalence of cognitive delay and SPPs for each dataset.

Second, we use multivariate regression analysis to examine the associations between different SPPs and infant cognitive developmental outcomes within the samples. Equations (1) and (2) define the specifications of the multivariate regressions that we use to demonstrate cognitive outcomes are associated with SPPs:

(1) $$Cog_{i}=\alpha +\beta_{1}SPP_{i}+\beta_{2}C_{i}+\beta_{3}H_{i}+\beta_{4}FE+\varepsilon$$

(2) $$Cog_{i}=\alpha_{4}+\gamma_{2}PP+\beta_{41}SES+\beta_{42}CE+\mathrm{Xi}\theta +\varepsilon$$

where $Cog_{i}\;$ stands for the cognitive score of infant i measured by the Bayley-III cognitive test, which is a continuous number that ranges from 49 to 145; $SPP_{i}$ is a dummy variable that indicates whether the caregiver did (or did not) engage in one of the *SPPs*; $C_{i}$ is infant *i*’s characteristics, including the infant’s age in months, the infant’s gender, and whether the infant is premature; $H_{i}$ is vector that includes the household characteristics, including mother’s age, mother’s educational attainment, household *SES*, and whether the household receives rural subsistence allowance (dibao); *FE* is province and year fixed effects. Standard errors are clustered at the village level.

Third and finally, we used multivariate regression analysis to examine the relationship between the frequency of *SPPs* and the study’s control variables:

(3) $$SPP=\alpha_{1}+\beta_{11}SES+\beta_{12}CE+X\;\theta +\varepsilon$$

where *SPP* denotes the frequency of *SPPs*, and *α* is the constant. In all equations, *SES* is socioeconomic status, and *CE* is mother’s educational attainment. In all equations we control for province and year fixed effects. Standard errors are clustered at the village level. The term $X_{i}$ is a vector of covariates that are included to capture characteristics of infants, such as gender, age, premature birth, and province, and *ε* is an error term for variance not captured by those variables in the model PP (which is a binary outcome, where 1 indicates presence of the specific *SPP*, and 0 indicates absence of such a practice), which represents three different measures of *SPP*: reading books, singing songs, and telling stories, each separately modeled. The regressors are indicated by $B_{ji}$, where *i* is the coefficient of regressor *i* in equation *j*.

In addition to our regression analysis, we also conduct a structural equation modeling (SEM) analysis to check for robustness. Our SEM analysis followed the guidelines of Schumacker and Lomax (2016) [27].

## 3. Results

### 3.1. Descriptive Characteristics

Table 1 shows the descriptive statistics of the infants and caregivers in our sample. This table comprises five studies with eight study sites for the survey respondents in five provinces. In total, the combined sample includes 4436 caregiver-infant/toddler dyads. The average age of the sampled infants across the eight study sites was 15 months, approximately half were male (52%), and only 5% of the sampled infants were born prematurely. The majority of primary caregivers were mothers (72%), and the average age of our sampled mothers was 28 years. These mothers had relatively lower levels of education, with the average level of educational attainment across all of the observations being junior high school (nine years of schooling). Within their communities, about 16% of households received a rural subsistence allowance.

### 3.2. Cognitive Scores

Table 2 presents the cognitive scores and rates of cognitive delays among infants and toddlers across the eight study sites (Columns 2–9). Row 1, Columns 2 to 9 present the average cognitive score of each dataset. On average, the infant cognitive score ranges from 103.8 to 83.7, with the weighted average across all pooled datasets being 95.6. Row 2, Columns 2 to 9 provide the average cognitive delay of the infants and toddlers in each study (for which an infant is counted as delayed if his or her score is below 95.4 and where delay is defined as a score that is less than −1 SD below the international mean). Of the 4436 sampled infants and toddlers, between 37% and 54% are measured as having cognitive delay. The weighted average across the pooled datasets shows that 51% of infants and toddlers suffer from cognitive delay (Row 2, Column 1). Rows 3 and 4 indicate that, out of the 51% of infants and toddlers with cognitive delay, an average of 30% are mildly delayed (with scores between 95.4 and 85.8), and as many as 21% are moderately to moderately/severely delayed (with scores below 85.8).

### 3.3. Stimulating Parenting Practices (SPPs)

Table 3 presents the findings for the prevalence of SPPs in which caregivers had engaged with their infants on the previous day (Columns 2–9). The results in Row 1 concern whether caregivers had read a book to their infant on the previous day. Across the pooled datasets, the results Columns 2 to 9 indicate that between 4% and 67% of caregivers had read a book to their infant, with the weighted average of the eight study sites indicating that only 22% of caregivers read a book to their infant. Rows 2 and 3 in Table 3 concern the prevalence of telling stories and singing songs with infants, respectively. As shown in Row 2, between 10% and 37% of caregivers told stories to their infants on the previous day. The weighted average of all datasets shows that only 17% of caregivers had told a story to their infant on the previous day (Column 1). Row 3 shows that between 2% and 64% of caregivers sang songs to their infants on the previous day. The weighted average across all datasets revealed that only 38% of caregivers sang songs to their infant on the previous day.

Row 4 of Table 3 presents the share of caregivers who engaged in any of the three SPPs measured. The results indicate that 39% to 70% of caregivers engaged in at least one SPP on the previous day, with the weighted average of all datasets showing that approximately half (49%) of caregivers engaged in at least one SPP on the previous day. The share of caregivers who engaged in multiple SPPs is considerably lower (where multiple is defined as engagement in two or more parenting practices), with the data showing that 8% to 41% of caregivers engaged in multiple SPPs on the previous day, with the weighted average across all datasets as revealing that only 19% of caregivers engage in multiple SPPs.

### 3.4. Correlates of Cognitive Development

Table 4 provides the correlations between individual SPP (reading books, telling stories, singing songs, engaging in at least one SPP, engaging in two or more SPPs) and infant cognitive development, holding household characteristics constant. Rows 1 to 3 indicate that reading books and singing songs are each significantly associated with an increase in infant cognitive score by 1.62 points (*p* = 0.003) and 2.00 points (*p* < 0.001), respectively. Telling stories to infants, however, is not significantly associated with infant cognitive scores.

Rows 6 to 9 of Table 4 provide the results of an examination of the relationships between caregiver and household characteristics and infant cognitive scores. Although whether the caregiver was the mother and mother’s age are not associated with infant cognitive scores, both mother’s educational attainment and the level of household assets are each significantly positively correlated with infant cognitive scores. Each additional year of mother’s educational attainment is associated with an increase in infant cognitive score by 0.33–0.38 points (*p* < 0.001). Whether a household has high assets is associated with an increase in infant cognitive score by 2.81–3.04 points (*p* < 0.001). The results also show that receiving a rural subsistence allowance is associated with a decrease of infant cognitive score by 1.63–1.67 points (*p* = 0.014/0.016).

### 3.5. Correlates of Stimulating Parenting Practices (SPPs)

Table 5 presents the correlations between SPPs and household characteristics (Columns 1–5). As seen in row 1, the primary caregiver as the mother is significantly associated with reading books to infants, telling stories to infants, singing songs to infants, engaging in at least one practice, and engaging in high levels of SPPs (*p* < 0.001). Row 2 shows that mother’s age is significantly associated with reading books to infants (*p* = 0.001), telling stories to infants (*p* < 0.001), and engaging in high levels of SPPs (*p* < 0.001). Similarly, mother’s educational attainment and higher household assets are associated with each SPP, engaging in at least one practice, and engaging in two or more SPPs (all *p*-values < 0.01). Surprisingly, Row shows that households’ receiving a rural subsistence allowance is not correlated with any of the three individual stimulating practices (Columns 1–3), engaging in one parenting practice, or engaging in two or more SPPs (Columns 4 and 5).

For robustness, we also re-ran the analysis that created the results reported in Table 4 and Table 5 using an SEM approach. The SEM results are reported in Appendix A Table A2. As can be seen from comparing the results from the SEM analysis with results in Table 4 and Table 5, we find that they are almost the same.

## 4. Discussion

The high rates of development delays found across our pooled dataset are consistent with other studies that examine infant development in rural China and exceed rates of cognitive delay found in many other LMICs. Across the five provinces, 51% of infants in our sample suffered from cognitive delay. These findings are consistent with other infant development studies conducted in rural China, which found rates of infant delay to be approximately three times higher than those found globally in healthy populations (approximately 15%) [28,29,30,31]. Further, rates of infant cognitive delay in our sample even exceeded rates of delay found in many LMICs. A developmental analysis, using the Early Childhood Development Index, found that, among 99,222 infants across 35 LMICs, the average rate of cognitive delay was approximately 36% [32]. Another study, which used national health surveys, found that, among 330,613 children across 63 LMICs, the average rate of cognitive delay was only 25% [33]. Our findings indicate that not only are average rates of cognitive delay in our rural China samples significantly higher than rates found in healthy populations but also are higher than rates of cognitive delay found in some LMICs.

The pooled dataset revealed one possible source of the high rates of delay: There are relatively low rates of SPPs across our pooled dataset when compared to other LMICs. Our study found that, on average, only 24% of caregivers had recently read to their infant, 25% had told stories to their infant, and 48% had sung to their infant in the past three days. In comparison, one study of parenting practices across 38 LMICs found that 19% of all surveyed caregivers had recently told stories to their infant, and an average of 56% had recently sung songs to their infant in the past three days [34]. Another study conducted across 39 LMICs found that an average of 22% of mothers had read to their infant in the past three days [35]. Given the high rates of cognitive delay found across our pooled dataset, the rates of SPPs in our study are low, even when compared to a subset of other LMICs.

When examining the relationship between infant cognitive development and SPPs, our study found that reading books and singing songs were highly correlated with infant cognitive outcomes, which suggests that these activities encourage high levels of caregiver-infant engagement. Multiple studies that examine the relationship between reading books and early cognitive development have found that reading books to young children fosters lifelong cognitive functions, regardless of family SES [36,37,38]. Similarly, studies that examine the relationship between singing songs to infants and cognitive outcomes have found that early exposure to singing is beneficial to general, lifelong cognitive outcomes [39,40,41].

In addition to finding significant associations between reading books, singing songs, and infant cognitive outcomes, we also found that both caregiver engagement in one SPP and caregiver engagement in two or more SPPs were significantly related to infant cognition. Surprisingly, engagement in one SPP was found to have a stronger association with infant cognitive development than with engagement in two or more SPPs, which may indicate that the quality of engagement is more important than the quantity. This theory aligns with a previous intervention study that was conducted in rural China, which found that improvement in the quality of parenting skills significantly increased infant development [42]. Although our study has shown that the quantity of caregiver investment in rural China is positively associated with infant cognitive outcomes, future studies should consider assessing the quality of engagement and its relationship to infant cognitive development.

Although there are other possible reasons behind high rates of delay, such as economic constraints or time constraints, evidence from recent studies in rural areas of China points to knowledge constraints as the main factor behind low levels of SPPs and high rates of developmental delays. Although rural households typically have fewer economic resources than urban households, recent studies have found that the majority of rural households have sufficient economic resources to invest in children’s development, and they have a desire to do so [10,43]. Moreover, in rural households in China, primary caregivers of young children typically do not work outside the home, meaning that unless they are caring for other family members (such as other children or elderly relatives), there are few time constraints that would prevent them from regularly engaging in SPPs with their children [43]. In contrast, multiple recent studies have found that rural caregivers do not have knowledge of how to effectively engage in SPPs, and rural caregivers often lack reliable sources of information about parenting [10,43,44], pointing to knowledge constraints as a primary cause for low levels of SPPs and high rates of delay.

Beyond examining SPPs and cognitive development, our analysis investigated the relationship between household characteristics, infant cognitive development, and SPPs. We found that whether the primary caregiver was the mother, mother’s age, and educational attainment were significantly positively associated with infant cognitive outcomes, engaging in all three SPPs (reading books, singing songs, telling stories), engaging in at least one practice, and engaging in two or more practices. These findings are consistent with the literature that shows that the education and age of caregivers are each positively associated with higher rates of parenting knowledge [45,46]. In turn, parenting knowledge has been linked to highly stimulating home environments, regardless of household SES [45,47]. Our findings indicate that, because our sampled mothers are less educated (junior high or lower), they may have limited exposure to parenting knowledge, and, thus, their engagement in SPPs is low. This also would suggest that future interventions specifically aimed at teaching caregivers essential parenting knowledge may be an effective way to increase SPP engagement, regardless of education or age.

## 5. Conclusions

This study is the first to examine large-scale developmental delays and SPPs across rural provinces in China. Our analysis of the parenting landscape in rural China used data from five studies with eight study sites, covering five rural provinces. Among our pooled sample of 4436 caregiver-toddler dyads, we examined the prevalence of infant cognitive delay, finding average rates of delay across these provinces to be higher than in many other LMICs. We also measured how SPPs and household characteristics relate to infant cognitive development. Finally, we assessed the relationship between caregiver and household characteristics and SPPs.

This paper has many strengths. It is the first to document rates of infant cognitive delay, SPPs, demographic characteristics, and their correlations across multiple datasets containing more than 4000 samples in rural China. The large sample size and the geographic range that our study covers provide evidence that cognitive delays are a problem across numerous rural communities in our sampled populations and provinces. This evidence can better inform policymakers about which types of families are at risk for high rates of cognitive delay and low SPPs and may be used to help inform community intervention projects.

We also acknowledge the limitations of this study. First, our study considers only cross-sectional data and is, therefore, unable to make causal claims. Second, given that China is an expansive and immensely diverse country with great regional disparities in indigenous culture and economic development, the five provinces in our study are not necessarily representative of all rural communities in China at a national level.

The results of this study suggest that increased investment in early childhood development and SPPs may have an important role in reducing cognitive delay in infants across rural China, which could be crucial to the future development of China’s economy. The literature shows that early cognitive delays have lifelong consequences, including lower levels of educational attainment and fewer career prospects and earnings [48,49]. On a larger scale, such widespread rates of cognitive delay are likely to hinder China’s human capital, which, in turn, could severely limit the future of China’s economy [50,51,52]. We recommend that future studies expand on ours by examining cognitive development and SPPs in rural communities in provinces that this study did not explore. Further research that identifies the extent of cognitive delays and SPPs across China will ultimately bring us closer to developing effective solutions and interventions for improving early childhood development across China’s most vulnerable populations.

## Figures and Tables

**Table 1:** Household and infant characteristics.

	(1)	(2)	(3)	(4)	(5)	(6)	(7)	(8)	(9)
	All	Shaanxi 2014	Yunnan 2015	Hebei 2015	Shaanxi 2016	Beijing 2017	Henan 2017	Shaanxi 2017	Yunnan 2019
	(*N* = 4436)	(*N* = 525)	(*N* = 210)	(*N* = 230)	(*N* = 1832)	(*N* = 81)	(*N* = 271)	(*N* = 273)	(*N* = 1014)
Panel A: household characteristics									
The primary caregiver is mother (1 = yes)	3179	350	164	185	1286	60	170	167	797
	0.72	0.67	0.78	0.80	0.70	0.74	0.63	0.61	0.79
Mother’s age (years)	27.5	26.42	26.81	29.15	27.98	28.52	27.62	28.61	26.55
	5.06	4.55	5.43	4.81	5.01	3.96	4.13	4.2	5.59
Mother’s years of schooling (years)	9.09	8.51	8.50	9.97	9.22	13.1	12.7	12.6	6.87
	3.56	2.53	3.10	2.94	2.64	2.51	3.08	2.85	4.14
Household has higher socioeconomic status (1 = yes)	2139	120	17	174	930	74	260	160	404
	0.48	0.23	0.081	0.76	0.51	0.91	0.96	0.59	0.40
Household receives rural subsistence allowance (dibao) (1 = yes)	831	145	31	24	202	6	19	33	371
	0.19	0.28	0.15	0.10	0.11	0.07	0.07	0.12	0.37
Panel B: infant characteristics									
Infant age (months)	16.3	24.5	11.7	13.1	14.5	18.2	20.3	18.8	15.3
	6.79	3.20	3.62	3.14	5.47	9.21	9.52	8.75	5.59
Male infant (1 = yes)	2284	251	111	125	944	44	152	133	524
	0.51	0.48	0.53	0.54	0.52	0.54	0.56	0.49	0.52
Infant is premature (1 = yes)	249	56	9	10	85	4	17	20	48
	0.06	0.11	0.04	0.04	0.05	0.05	0.06	0.07	0.05

Notes: Data are frequency and percent for binary variables (1 = yes; 0 = no), and mean and standard deviation for numeric variables (years or months).

**Table 2:** Prevalence of cognitive delay.

	(1)	(2)	(3)	(4)	(5)	(6)	(7)	(8)	(9)
	All	Shaanxi 2014	Yunnan 2015	Hebei 2015	Shaanxi 2016	Beijing 2017	Henan 2017	Shaanxi 2017	Yunnan 2019
	(*N* = 4436)	(*N* = 525)	(*N* = 210)	(*N* = 230)	(*N* = 1832)	(*N* = 81)	(*N* = 271)	(*N* = 273)	(*N* = 1014)
Cognitive score	95.62	83.72	95.93	100.61	95.91	98.70	103.84	98.42	96.87
	15.02	20.78	12.61	12.02	12.57	12.54	13.77	16.74	13.15
Cognitive delay (1 = yes)	2225	221	119	93	988	38	101	139	526
	0.50	0.42	0.57	0.40	0.54	0.47	0.37	0.51	0.52
Mild delay (1 = yes)	1247	81	73	61	538	24	78	78	314
	0.28	0.15	0.35	0.27	0.29	0.30	0.29	0.29	0.31
Moderate-severe delay (1 = yes)	978	140	46	32	450	14	23	61	212
	0.22	0.27	0.22	0.14	0.25	0.17	0.08	0.22	0.21
Moderate-severe delay (1 = yes)	978	140	46	32	450	14	23	61	212
	0.22	0.27	0.22	0.14	0.25	0.17	0.08	0.22	0.21

Notes: ^a^ Data are frequency and percent for binary variables (1 = yes; 0 = no), and mean and standard deviation for numeric variables (years or months). ^b^ The mean score (standard deviation) of Bayley III scales of Infant Development (BSID-III) is expected to be 105 (9.6) for the cognitive scale in a healthy population. Mild delay is defined as a score between one and two SDs below the mean. This translates to a score between 85.8 (inclusive) and 95.4 on the cognitive scale. A moderate or severe delay is defined as a score that is more than two SDs below the mean, which is below 85.8 for the cognitive scale. ^c^ Bayley I Scales of Infant Development (BSID-I) was used for collecting data from Shaanxi in 2014 (Column2). For BSID-I, mild delay is defined as a score between 70 (inclusive) and 80 on the cognitive scale; A moderate or severe delay is defined as a score below 70 for the cognitive scale.

**Table 3:** Prevalence of stimulating parenting practices.

	(1)	(2)	(3)	(4)	(5)	(6)	(7)	(8)	(9)
	All	Shaanxi 2014	Yunnan 2015	Hebei 2015	Shaanxi 2016	Beijing 2017	Henan 2017	Shaanxi 2017	Yunnan 2019
	(*N* = 4436)	(*N* = 525)	(*N* = 210)	(*N* = 230)	(*N* = 1832)	(*N* = 81)	(*N* = 271)	(*N* = 273)	(*N* = 1014)
Read book to infant (1 = yes)	960	21	107	155	384	25	78	64	126
	0.22	0.04	0.51	0.67	0.21	0.31	0.29	0.23	0.12
Told story to infant (1 = yes)	742	57	20	42	338	30	66	71	118
	0.17	0.11	0.10	0.18	0.18	0.37	0.24	0.26	0.12
Sang song to infant (1 = yes)	1678	195	5	20	795	52	135	115	361
	0.38	0.37	0.02	0.09	0.43	0.64	0.50	0.42	0.36
At least one practice (1 = yes)	2168	207	113	162	923	58	157	130	418
	0.49	0.39	0.54	0.70	0.50	0.72	0.58	0.48	0.41
Two or more practices (1 = two or more practices)	848	50	17	36	414	33	79	78	141
	0.19	0.10	0.08	0.16	0.23	0.41	0.29	0.29	0.14

Notes: ^a^ Data are frequency and percent. ^b^ The simulating parent practices (read book to infant, told story to infant, and sang song to infant) reported in columns 2–5 measured caregiver engagement in the previous day; the columns 6–9 measured caregiver engagement in the past three days.

**Table 4:** Correlations between stimulating parenting practices and infant cognitive scores.

	(1)			(3)			(3)			(4)			(5)		
	Dependent Variable: Infant Cognitive Score														
	β	95% CI	*p*	β	95% CI	*p*	β	95% CI	*p*	β	95% CI	*p*	β	95% CI	*p*
Read book to infant (1 = yes)	1.62 **	0.56, 2.69	0.003												
Told story to infant (1 = yes)				1.08	−0.06, 2.21	0.062									
Sang song to infant (1 = yes)							2.00 ***	0.95, 3.05	<0.001						
At least one practice (1 = yes)										2.35 ***	1.38, 3.32	<0.001			
Two or more practices (1 = yes)													1.30 *	0.20, 2.40	0.020
The primary caregiver is mother	−0.73	−1.78, 0.32	0.174	−0.63	−1.67, 0.42	0.238	−0.91	−1.95, 0.13	0.088	−1.01	−2.06, 0.03	0.057	−0.68	−1.73, 0.37	0.203
Mother’s age (years)	0.06	−0.03, 0.14	0.169	0.06	−0.02, 0.14	0.159	0.06	−0.02, 0.15	0.143	0.06	−0.02, 0.15	0.145	0.06	−0.03, 0.14	0.172
Mother’s years of schooling (years)	0.37 ***	0.22, 0.52	<0.001	0.38 ***	0.23, 0.53	<0.001	0.34 ***	0.19, 0.49	<0.001	0.33 ***	0.17, 0.48	<0.001	0.37 ***	0.23, 0.52	<0.001
Household has higher socioeconomic status (1 = yes)	3.01 ***	2.07, 3.94	<0.001	3.04 ***	2.10, 3.98	<0.001	2.84 ***	1.93, 3.76	<0.001	2.81 ***	1.90, 3.72	<0.001	3.00 ***	2.07, 3.94	<0.001
Household receives rural subsistence allowance (dibao) (1 = yes)	−1.66 *	−2.98, −0.34	0.014	−1.64 *	−2.97, −0.32	0.015	−1.66 *	−2.99, −0.32	0.015	−1.63 *	−2.96, −0.31	0.016	−1.67 *	−3.00, −0.34	0.014
Adjusted R-squared	0.12			0.12			0.13			0.13			0.12		
Number of observations	4434			4434			4433			4433			4434		

Notes: ^a^ Cognitive score was measured using the Bayley-III scale and Bayley I Scales of Infant Development (BSID-I). Scores in this sample ranged from 49 to 145. ^b^ Results of the ordinary least squares model, controlling for province and year fixed effect, are reported; standard errors are clustered at the village level. ^c^ Logistic regressions also adjust for three infant characteristics: infant age (months), male infant (1 = yes), and infant premature (1 = yes). ^d^ A generalized structural equation model (GSEM) was used to test robustness of measures and can be found in Appendix A Table A2. ^e^ * *p* < 0.05; ** *p* < 0.01; *** *p* < 0.001. ^f^ Regression 1 examines the correlation between infant cognition and reading books, holding household characteristics constant. In the next four regressions (2–5) we look at the correlation between infant cognition and telling stories/singing songs/engaging in at least one SPP/engaging in two or more SPPs (respectively). All household characteristics are held constant. ^g^ A generalized structural equation model (GSEM) was used to test robustness of measures and can be found in Appendix A Table A2.

**Table 5:** Correlations between stimulating parenting practices and household characteristics.

	(1)			(2)			(3)			(4)			(5)		
	Read Book to Infant			Told Story to Infant			Sang Song to Infant			At Least One Practice			Two or More Practices		
	β	95% CI	*p*	β	95% CI	*p*	β	95% CI	*p*	β	95% CI	*p*	β	95% CI	*p*
The primary caregiver is mother	0.09 ***	0.07, 0.12	<0.001	0.04 ***	0.02, 0.07	0.001	0.16 ***	0.13,0.19	<0.001	0.18 ***	0.15, 0.21	<0.001	0.08 ***	0.05, 0.11	<0.001
Mother‘s age (years)	0.004 **	0.001 0.006	0.001	0.004 **	0.001 0.006	0.002	0.001	−0.002 0.004	−0.366	0.001	−0.002 0.004	0.514	0.005 ***	0.002 0.007	<0.001
Mother’s years of schooling (years)	0.02 ***	0.01, 0.02	<0.001	0.01 ***	0.01, 0.02	<0.001	0.03 ***	0.02, 0.03	<0.001	0.03 ***	0.02, 0.03	<0.001	0.02 ***	0.01, 0.02	<0.001
Household has higher socioeconomic status (1 = yes)	0.07 ***	0.04, 0.09	<0.001	0.07 ***	0.04, 0.10	<0.001	0.11 ***	0.08, 0.14	<0.001	0.12 ***	0.09, 0.16	<0.001	0.08 ***	0.06, 0.11	<0.001
Household receives rural subsistence allowance (*dibao*) (1 = yes)	0.00	−0.03, 0.03	0.864	−0.02	−0.06, 0.02	0.375	0.01	−0.02, 0.04	0.589	−0.01	−0.04, 0.02	0.516	0.01	−0.03, 0.04	0.638
Number of observations	4434			4434			4433			4434			4434		

Notes: ^a^ Logistic regressions are used and marginal effects are reported in model (1) to (5). ^b^ Logistic regressions control for provinces and years fixed effects; standard errors are clustered at the village level. ^c^ Logistic regressions also adjust for three infant characteristics: infant age (months), male infant (1 = yes), and infant premature (1 = yes). ^d^ ** *p* < 0.01; *** *p* < 0.001. ^e^ The coefficient and 95% CI of Mother’s age (years) are shown in three decimal digits because all of them are smaller than 0.01. ^f^ A generalized structural equation model (GSEM) was used to test robustness of measures and can be found in Appendix A Table A2.

## Data Availability

The Stata code and data permissible for sharing will be made available upon request from H.X. (haoxue@stanford.edu).

## Appendix A

**Table A1:** Summary of studies included in the pooled dataset.

Study	Location of the Study	Date	Corresponding Publication
Study 1	Shaanxi	2014	Sylvia, S., Warrinnier, N., Luo, R., Yue, A., Attanasio, O., Medina, A., Rozelle, S. From quantity to quality: delivering a home-based parenting intervention through China’s family planning cadres. *Econ. J.* **2021**, *131*, 1365–1400. doi:10.1093/ej/ueaa114 [42].
Study 2a	Yunnan	2015	Luo, R., Jia, F., Yue, A., Zhang, L., Lyu, Q., Shi, Y., Yang, M., Medina, A., Kotb, S., Rozelle, S. Passive parenting and its association with early child development. *Early Child Dev. Care.* **2017**, *189*, 1–15. doi:10.1080/03004430.2017.1407318 [53].
Study 2b	Hebei	2015	
Study 3	Shaanxi	2016	Zhong, J., He, Y., Gao, J., Wang, T., Luo, R. Parenting knowledge, parental investments, and early childhood development in rural households in western China.” *Int. J. Environ. Res. Public Health.* **2020**, *17*, 2792 [54].
Study 4a	Beijing	2017	Wang, L., Liang, W., Zhang, S., Jonsson, L., Li, M., Yu, C., Sun, Y., Ma, Q., Bai, Y., Abbey, C., et al. Are infant/toddler developmental delays a problem across rural China? *J. Comp. Econ.* **2019**, *47*, 458–469. doi: 10.1016/j.jce.2019.02.003 [55].
Study 4b	Henan	2017	
Study 4c	Shaanxi	2017	
Study 5	Yunnan	2019	Zhang, S., Wang, L., Xian, Y., Bai, Y. Mental health issues among caregivers of young children in rural China: prevalence, risk factors, and links to child developmental outcomes. *Int. J. Environ. Res. Public Health.* **2021**, *18*, 197 [56].

**Table A2:** Correlations between infant cognitive scores, stimulating parenting practices and household characteristics using generalized structural equation model (GSEM).

Variables	Coefficient	95% CI	*p*-Value
Panel A Dependent variable: infant cognitive score			
Read book to infant (1 = yes)	2.67 ***	1.50, 3.84	<0.001
Told story to infant (1 = yes)	−0.25	−1.39, 0.89	0.670
Sang song to infant (1 = yes)	1.12 *	0.07, 2.16	0.036
The primary caregiver is mother	−1.31 *	−2.34, −0.29	0.012
Mother’s age (years)	0.11 *	0.02, 0.20	0.015
Mother’s years of schooling	0.34 ***	0.16, 0.51	<0.001
Household has higher socioeconomic status (1 = yes)	4.35 ***	3.34, 5.35	<0.001
Household receives rural subsistence allowance (dibao) (1 = yes)	−1.57 *	−3.08, −0.06	0.042
Panel B Dependent variable: Read book to infant (1 = yes)			
The primary caregiver is mother	0.10 ***	0.06, 0.13	<0.001
Mother’s age (years)	0.01 ***	0.00, 0.01	<0.001
Mother’s years of schooling	0.02 ***	0.01, 0.02	<0.001
Household has higher socioeconomic status (1 = yes)	0.07 ***	0.04, 0.10	<0.001
Household receives rural subsistence allowance (dibao) (1 = yes)	−0.03	−0.07, 0.00	0.055
Panel C Dependent variable: Told story to infant (1 = yes)			
The primary caregiver is mother	0.04 **	0.02, 0.06	0.001
Mother’s age (years)	0.00 ***	0.00, 0.01	<0.001
Mother’s years of schooling	0.01 ***	0.01, 0.02	<0.001
Household has higher socioeconomic status (1 = yes)	0.08 *	0.05, 0.11	0.05
Household receives rural subsistence allowance (dibao) (1 = yes)	−0.03	−0.06, 0.01	0.16
Panel D Dependent variable: Sang song to infant (1 = yes)			
The primary caregiver is mother	0.16	0.12, 0.19	0.12
Mother’s age (years)	0.00 ***	0.00, 0.00	<0.001
Mother’s years of schooling	0.02 ***	0.02, 0.03	<0.001
Household has higher socioeconomic status (1 = yes)	0.12 ***	0.09, 0.16	<0.001
Household receives rural subsistence allowance (dibao) (1 = yes)	0.02	−0.02, 0.05	0.30

Notes: ^a^ Generalized structural equation model (GSEM) is used to estimate the correlation between infant cognitive scores, stimulating parenting practices and household characteristics. Standard errors account for clustering at the village level. ^b^ The GSEM also adjusts for three infant characteristics: infant age (months), male infant (1 = yes), and infant premature (1 = yes). ^c^ Logistic regressions are used, and marginal effects are reported in Panel B, C, and D. ^d^ * *p* < 0.05; ** *p* < 0.01; *** *p* < 0.001.
